# Integrated Relaxation Pressure (IRP) Distinguishes between Reflux-Predominant and Dysphagia-Predominant Phenotypes of Esophageal “Absent Contractility”

**DOI:** 10.3390/jcm11216287

**Published:** 2022-10-25

**Authors:** Daniel L. Cohen, Anton Bermont, Vered Richter, Narjes Azzam, Haim Shirin, Ram Dickman, Amir Mari

**Affiliations:** 1 The Gonczarowski Family Institute of Gastroenterology and Liver Diseases, Shamir (Assaf Harofeh) Medical Center, Zerifin 703000, Israel; 2 Gastroenterology and Endoscopy Unit, Nazareth EMMS Hospital, Nazareth 16100, Israel; 3 Sackler School of Medicine, Tel Aviv University, Tel Aviv 6997801, Israel; 4 Division of Gastroenterology, Rabin Medical Center, Beilinson Hospital, Petach Tikva 4941492, Israel; 5 Faculty of Medicine, Bar Ilan University, Safed 1311502, Israel

**Keywords:** esophageal motility disorders, manometry, dysphagia, gastroesophageal reflux, achalasia, deglutition disorders

## Abstract

Background: Patients with absent contractility (AC) often suffer from either reflux or dysphagia. It remains unclear what factors determine which phenotype patients present with. We sought to evaluate if high-resolution manometry metrics, especially integrated relaxation pressure (IRP), could explain this. Methods: Cases of AC from three medical centers were reviewed for demographic, clinical, and manometric data. Cases with an IRP between 10–15 mmHg or subsequent diagnosis of achalasia were excluded. Results: 69 subjects were included (mean age 56.1; 71% female). A total of 41 (59.4%) were reflux-predominant. The reflux-predominant group was younger (51.1 vs. 63.5, *p* = 0.002) and had lower median LES basal pressures (7.5 vs. 12.5 mmHg, *p* = 0.014) and IRP values (1.5 vs. 5.6 mmHg, *p* < 0.001) compared to the dysphagia group. When divided into tertiles, the trend in symptoms between LES basal pressure tertiles was not significant. However, the trend for IRP was significant (*p* < 0.001). For example, in the lowest IRP tertile, 91.3% of subjects were reflux-predominant compared to only 26.1% in the highest tertile, while the dysphagia-predominant group increased from 8.7% to 73.9%. In a regression model controlling for age and using IRP tertile 1 as the reference, having an IRP in tertile 2 increased the likelihood of having dysphagia-predominant disease by 7, while being in tertile 3 increased the likelihood by 22. Conclusions: IRP helps distinguish between the reflux-predominant and dysphagia-predominant phenotypes of AC. This may have therapeutic clinical consequences as procedures such as fundoplication to tighten the LES may benefit patients with reflux and a low IRP, while procedures like peroral endoscopic myotomy (POEM) to disrupt the LES may benefit patients with dysphagia and a relatively high IRP.

## 1. Introduction

Absent contractility (AC) is an esophageal motility disorder defined as a normal integrated residual pressure (IRP) with 100% failed peristalsis [1]. It has been associated with rheumatologic diseases such as systemic sclerosis and was previously referred to as “scleroderma esophagus [2]”.

The most common presenting symptoms of AC are reflux symptoms or dysphagia [3,4,5,6,7]. Reflux is felt to be common due to a normotensive or hypotensive lower esophageal sphincter (LES) allowing for reflux, combined with absent peristalsis in the esophageal body allowing for the refluxate to remain in the esophagus. Therefore, for many, the “typical” patient with AC has scleroderma and significant reflux disease [8]. However, some patients do not report reflux symptoms and instead complain of dysphagia. It is not clear why patients predominantly have one symptom or the other.

These different phenotypes of AC may be explained by differences in LES residual pressure as measured by IRP. In a study of AC patients with dysphagia undergoing peroral endoscopic myotomy (POEM), the IRP was 10.8 mmHg [9], while in a study of AC patients with reflux undergoing fundoplication, the LES residual pressure was 0.8 mmHg [10]. While both of these values are within the normal range of IRP (<15 mmHg) [1], we hypothesized that higher residual LES pressures may explain dysphagia symptoms while lower LES residual pressures may explain a predilection for acid reflux. To date, no study has performed a head-to-head comparison of manometric values, including IRP, between these two phenotypes of AC patients.

Therefore, the aim of this study was to evaluate if there are differences in manometric values, especially IRP, between reflux-predominant and dysphagia-predominant patients with AC.

## 2. Materials and Methods

### 2.1. Study Design and Subjects

Cases of AC were identified retrospectively by reviewing the esophageal high-resolution manometry (HRM) studies performed at three tertiary medical centers in Israel. This cohort of patients has previously been described [3]. The study was approved by the Institutional Review Board (ASF-155-22).

All HRM studies were reviewed by experts in esophageal motility to confirm the diagnosis of AC as per the Chicago classification, version 4.0 [1]. As an IRP between 10–15 mmHg can be found in Type I achalasia patients, these cases were excluded as has been recommended in guidelines [1]. Additionally, any patient who was subsequently diagnosed with achalasia was excluded.

Importantly, all patients included in the study previously underwent upper endoscopy. Some of these endoscopies were performed at other medical centers and patients were only referred to our centers for HRM testing. All patients with pathological findings on an endoscopy that might explain dysphagia, such as eosinophilic esophagitis, were excluded.

Data on demographics, clinical symptoms, and manometric variables were obtained via chart review. The main manometric variables assessed were the LES basal pressure and the IRP. Any case with an incomplete HRM study or missing clinical data was excluded.

Subjects were divided into two groups (reflux and dysphagia) based on the predominant presenting symptom on their HRM referral form. This was confirmed by reviewing each subject’s medical chart. Symptoms of heartburn, regurgitation, and vomiting were included in the reflux group, while symptoms of dysphagia and difficulty swallowing comprised the dysphagia group. The groups were compared to each other with the primary end point being differences in the IRP value between the groups.

### 2.2. HRM Protocol

HRM studies were performed using the ManoScan system (Medtronic, Minneapolis, MN, USA). All manometries were performed according to the standard protocol at our institutions in which 10 wet swallows were completed after successful placement of the catheter beyond the esophagogastric junction. All HRM were analyzed by senior gastroenterologists who are experts in the field of Neurogastroenterology. Rapid Drinking challenge (RDC) with 200 mL water was performed in only one of the participating centers.

### 2.3. Statistical Analysis

Categorical variables were summarized as frequency and percentage. T squared test and Fisher’s exact test were applied to compare categorical variables between the groups. LES basal pressure and IRP were evaluated for normal distribution using histogram, and since they were skewed, they were reported as median and interquartile range (IQR). Mann-Whitney tests were used to compare these variables between the groups. For further analyses, these variables were divided into tertiles and Pearson Chi Squared tests were performed. Finally, multiple logistic regression was used to study the association between IRP and symptom phenotype while controlling for age.

All statistical tests were two-sided and *p* < 0.05 was considered as statistically significant. SPSS software was used for all statistical analysis (IBM SPSS Statistics for Windows, version 26, IBM Corporation, Armnok, NY, USA, 2019).

## 3. Results

### 3.1. Characteristics of the Overall Cohort of Absent Contractility Patients

**Figure 1:**
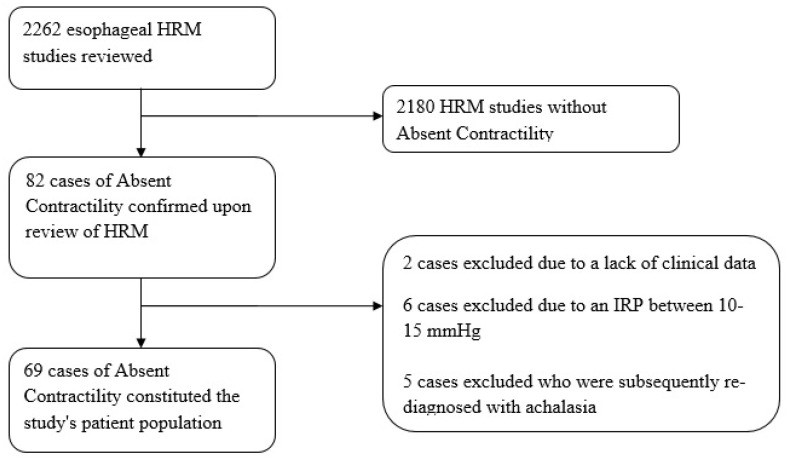
Flowchart of Absent Contractility patients. IRP: integrated relaxation pressure; HRM: high-resolution manometry.

From a total of 2262 HRM studies, 82 (3.6%) were found to have AC. Of these, 2 were excluded due to a lack of clinical data, 6 were excluded due to an IRP between 10–15 mmHg, and 5 were excluded as they were subsequently re-diagnosed with achalasia during follow-up (mean follow-up time of 14 months). Thus, a total of 69 subjects were included in this study (Figure 1).

Details of the study population can be found in Table 1. The mean age was 56.1 years old and 71% were women. Sixteen (23.2%) subjects had underlying rheumatologic disease including 6 (8.7%) with systemic sclerosis.

The most common findings on upper endoscopy included a hiatal hernia (40.6%), reflux esophagitis (35.9%), and a normal exam (32.8%). On HRM, the median LES basal pressure was 9.5 mmHg with an IRP of 2.4 mmHg. Additionally, RDC was performed during HRM for 21 patients (all performed at one of the participating medical centers since 2018). None of the RDCs detected esophageal pressurization, but 12 of 21 (57.1%) provoked the patient’s symptoms, particularly dysphagia. Only 3 of 21 (14.2%) RDCs showed improved contractility of esophageal peristalsis indicating some degree of esophageal motor reserve.

### 3.2. Differences between Reflux-Predominant and Dysphagia-Predominant Subjects

From the overall cohort, 41 subjects (59.4%) had reflux-predominant symptoms while 28 (40.6%) were of the dysphagia-predominant phenotype. 

Several differences were noted between these groups (Table 1). The reflux-predominant group was more likely to be younger (51.1 vs. 63.5 years old, *p* = 0.002). There was a trend for them to be more likely to have rheumatologic diseases (29.2% vs. 14.3%, *p* = 0.148) and they were more likely to have Raynaud’s phenomenon (22.0% vs. 3.6%, *p* < 0.041). As expected, the reflux-predominant group was also more likely to have reflux esophagitis and less likely to have a normal endoscopy.

**Figure 2:**
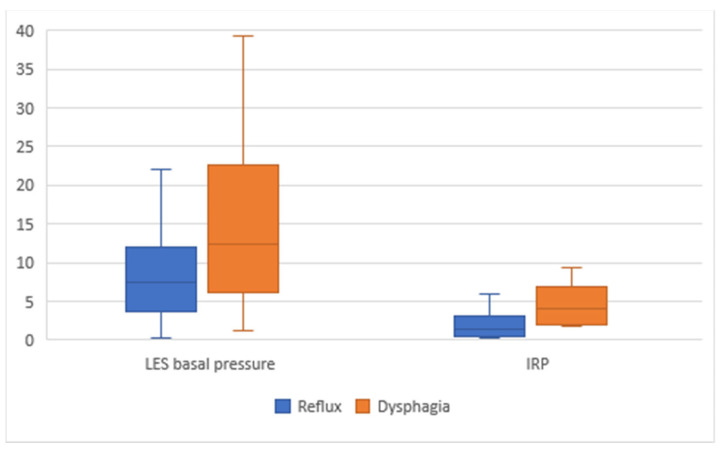
HRM Metrics Based on Absent Contractility Phenotype.

In terms of HRM metrics, the reflux-predominant group had significantly lower LES basal pressures (7.5 vs. 12.5 mmHg, *p* = 0.014) and IRP values (1.5 vs. 5.6 mmHg, *p* < 0.001) compared to the dysphagia-predominant group (Table 1, Figure 2).

### 3.3. Further Analyses of HRM Metrics by Dividing the Cohort into Tertiles

Further analyses were performed by dividing the cohort of 69 subjects into three tertiles. First, the cohort was divided into tertiles based on LES basal pressures (Table 2A). This analysis showed that as the LES basal pressure increased, the percentage of subjects with reflux decreased while the percentage with dysphagia increased, although this trend was not significant (*p* = 0.108).

**Figure 3:**
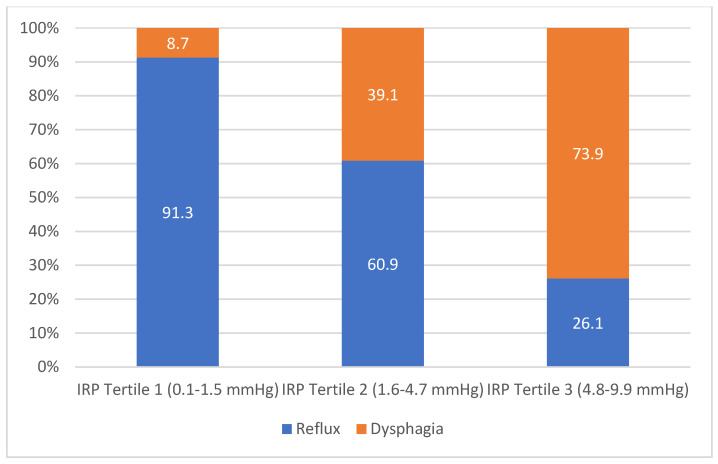
IRP Values are Associated with Symptom Phenotype (*p* < 0.001).

However, the findings were more striking when evaluating IRP values (Table 2B and Figure 3). In the lowest IRP tertile, 91.3% of subjects had reflux-predominant disease while this percentage decreased to just 26.1% in the highest IRP tertile. The opposite also occurred as the dysphagia-predominant group increased from 8.7% to 73.9% from tertile 1 to tertile 3. These findings were significant (*p* < 0.001).

### 3.4. Regression Analyses of the Effect of IRP on Symptom Phenotype

Finally, given the significance of IRP, a multivariate regression analysis model was constructed controlling for age to quantify the effect of IRP (Table 3). Using IRP tertile 1 as the reference, this analysis found that having an IRP in tertile 2 increased the likelihood of having dysphagia-predominant disease by an odds ratio (OR) of 7.035, while being in tertile 3 increased the likelihood by an OR of 22.188.

## 4. Discussion

This is the first study to evaluate the relationship between HRM metrics and symptom phenotypes in patients with AC. We found that even though the IRP values in AC are within the normal limits [1], IRP was significantly associated with the different phenotypes of AC, with lower IRP values being found in reflux-predominant disease and higher IRP values being seen in dysphagia-predominant disease.

AC is an uncommon disorder found in 5–7.1% of patients with non-obstructive dysphagia [11,12]. The term AC only dates back to the third version of the Chicago classification from 2015 [13]. Prior to that, it was referred to as “absent peristalsis” in the earlier versions of the Chicago classification [14,15], and “scleroderma esophagus” or “scleroderma-like esophagus” in conventional manometry [2].

In many ways, AC is equivalent to treated type I achalasia as both conditions have the same manometric findings. As such, reviewing the data on treated achalasia patients may be helpful as they may serve as a model for understanding patients with AC. Given their similar manometric findings, one would expect that they should have similar symptoms. When achalasia is successfully treated, numerous studies have shown that dysphagia symptoms improve remarkably [16]. However, after treatment of achalasia, reflux symptoms often increase and many patients require medication to treat GERD [16]. From this comparison, one would expect that much like successfully-treated achalasia patients, patients with AC should be more likely to suffer from reflux than dysphagia. Indeed, our study found that a majority of AC patients had reflux-prominent disease (~60%).

Some achalasia patients, even after treatment, continue to have dysphagia symptoms. There are several different examinations which can be performed to evaluate persistent symptoms in these patients. While newer tests, such as a timed barium esophogram (TBE) or functional luminal imaging probe, have been described, the most studied test is manometry. Studies of patients undergoing pneumatic dilation have shown that symptoms are likely to improve if the post-treatment IRP is less than 10 mmHg on manometry [17,18].

In our study, rather than using a cutoff value, we investigated the absolute IRP value as all of our subjects had an IRP < 10 mmHg. We found that higher IRP values, even though still within the accepted normal range, were associated with dysphagia, while lower IRP values were associated with reflux. This suggests that “normal” IRP values may indeed cause dysphagia symptoms in the setting of an esophagus lacking peristalsis.

Our findings have potential clinical significance and may affect the therapeutic options available to patients with AC. Surgical fundoplication is often performed for treatment of difficult-to-treat reflux disease. While the absence of esophageal peristalsis is a relative contraindication to surgery due to the fear of dysphagia developing post-operatively, several studies have already shown that fundoplication can be performed to treat reflux in AC patients with good clinical results [10,19,20,21].

For AC patients with dysphagia and a relatively high IRP, POEM may be a therapeutic option, although there is currently a paucity of data to support this. In a recent small study, six patients with AC underwent POEM and had a decrease in IRP values and Eckardt scores, although the improvement in Eckardt scores was less than patients with achalasia or EGJ Outflow Obstruction who underwent POEM [9].

We do not mean to suggest that IRP alone should be used to determine which therapy to offer a patient, especially when considering invasive treatments such as surgery. A global view of the patient needs to be taken including their symptoms, overall health, endoscopic findings, manometric data including IRP, and response to other treatments such proton-pump inhibitors, as well as local expertise.

Our study has some limitations. It is a retrospective study and contains a relatively small number of patients. We divided the subjects into two groups (reflux and dysphagia) based on their main symptoms, but some subjects may have had both of these symptoms to a certain degree, and the predominant symptom may also have changed over time. Symptoms were taken from a review of charts, and not prospectively collected, nor was a standardized symptom score such as the Eckardt score used. Additionally, for this descriptive observational study, we also do not have any follow-up manometric data assessing whether values such as IRP remain stable over time, or how they respond to interventions such as POEM or fundoplication. Further, we were unable to correlate how HRM metrics such as IRP may relate to findings on complementary tests such as TBE in patients with AC. Despite these limitations, this study benefits from its multicenter design and use of standard HRM metrics.

## 5. Conclusions

In conclusion, this study has shown that IRP is useful in distinguishing between the reflux-predominant and dysphagia-predominant phenotypes of AC. This suggests that slight differences in LES relaxation pressure, even within the reported normal limits, can have real clinical significance. Our findings may also affect treatment of patients with AC as procedures to tighten the LES, such as a fundoplication, may benefit AC patients with reflux and a low IRP, while procedures to disrupt the LES, such as POEM, may help AC patients with dysphagia and a relatively elevated IRP. Future studies assessing outcomes of these treatments in patients with AC are warranted.

## Figures and Tables

**Table 1:** Comparison between Reflux-predominant and Dysphagia-predominant Patients with Absent Contractility.

	Overall	Reflux-Predominant	Dysphagia-Predominant	*p*-Value
	n = 69	n = 41 (59.4%)	n = 28 (40.6%)	
Demographics				
Age (years, SD)	56.1 (16.6)	51.1 (15.6)	63.5 (15.5)	**0.002**
Female Gender	49 (71.0%)	28 (68.3%)	21 (75.0%)	0.546
Pre-existing medical conditions				
Alcohol use	6 (8.7%)	4 (9.8%)	2 (7.1%)	1.000
Tobacco use	22 (31.8%)	14 (34.1%)	8 (28.6%)	0.626
Diabetes	11 (15.9%)	7 (17.1%)	4 (14.3%)	1.000
Thyroid disease	8 (11.6%)	4 (9.8%)	4 (14.3%)	0.706
Rheumatalogic disease	16 (23.2%)	12 (29.2%)	4 (14.3%)	0.148
Systemic sclerosis	6 (8.7%)	6 (14.6%)	0 (0%)	0.074
Raynaud’s phenomenon	10 (14.5%)	9 (22.0%)	1 (3.6%)	**0.041**
Prior gastric surgery	11 (15.9%)	5 (12.2%)	6 (21.4%)	0.334
Baseline endoscopy findings	n = 64	n = 37	n = 27	
Normal	21 (32.8%)	8 (21.6%)	13 (48.1%)	**0.026**
Hiatal hernia	26 (40.6%)	17 (45.9%)	9 (33.3%)	0.310
Reflux esophagitis	23 (35.9%)	17 (45.9%)	6 (22.2%)	0.051
Barrett’s esophagus	3 (4.7%)	3 (8.1%)	0 (0%)	0.257
Candida esophagitis	3 (4.7%)	2 (5.4%)	1 (3.7%)	1.000
Retained food	3 (4.7%)	1 (2.7%)	2 (7.4%)	0.568
Dilated esophagus	9 (14.1%)	6 (16.2%)	3 (11.1%)	0.722
Epiphrenic diverticulum	2 (3.1%)	1 (2.7%)	1 (3.7%)	1.000
HRM Metrics				
LES basal pressure (mmHg, IQR)	9.5 (4.2–15.2)	7.5 (3.6–12.1)	12.5 (6.2–22.7)	**0.014**
IRP (mmHg, IQR)	2.4 (1.1–5.9)	1.5 (0.5–3.1)	5.6 (2.1–7.1)	**<0.001**

SD: standard deviation; HRM: high-resolution manometry; LES: lower esophageal sphincter; IRP: integrated relaxation pressure; IQR: inter-quartile range. *p*-values in bold represent statistically significant differences.

**Table 2:** Prevalence of Absent Contractility Phenotypes according to HRM Metrics.

A. LES Basal Pressure	Reflux	Dysphagia	*p*-Value
Tertile 1 (0.1–5.4 mmHg)	17 (73.9%)	6 (26.1%)	*p* = 0.108
Tertile 2 (5.5–12.7 mmHg)	14 (60.9%)	9 (39.1%)	
Tertile 3 (12.8–39.1 mmHg)	10 (43.5%)	13 (56.5%)	
**B. IRP**	**Reflux**	**Dysphagia**	
Tertile 1 (0.1–1.5 mmHg)	21 (91.3%)	2 (8.7%)	***p* < 0.001**
Tertile 2 (1.6–4.7 mmHg)	14 (60.9%)	9 (39.1%)	
Tertile 3 (4.8–9.9 mmHg)	6 (26.1%)	17 (73.9%)	

HRM: high-resolution manometry; LES: lower esophageal sphincter; IRP: integrated relaxation pressure. *p*-values in bold represent statistically significant differences.

**Table 3:** Logistic regression Analysis Evaluating the Effect of IRP on the Likelihood of Dysphagia-predominant Disease.

	Odds Ratio	Confidence Interval	*p*-Value
IRP Tertile 1	Reference	--	--
IRP Tertile 2	7.035	1.264–39.157	*p* = 0.026
IRP Tertile 3	22.188	3.821–128.833	*p* = 0.001

IRP: integrated relaxation pressure.

## Data Availability

The database is available from the corresponding author upon reasonable request.

## Abbreviations

IRP	integrated relaxation pressure
AC	absent contractility
LES	lower esophageal sphincter
POEM	peroral endoscopic myotomy
HRM	high-resolution manometry
RDC	rapid drink challenge
IQR	inter-quartile range
SD	standard deviation
OR	odds ratio
TBE	timed barium esophogram
